# Simultaneous visualization of different genomes (J, JSt and St) in a *Thinopyrum intermedium* × *Thinopyrum ponticum* synthetic hybrid (Poaceae) and in its parental species by multicolour genomic in situ hybridization (mcGISH)

**DOI:** 10.3897/CompCytogen.v10i2.7305

**Published:** 2016-06-17

**Authors:** Klaudia Kruppa, Márta Molnár-Láng

**Affiliations:** 1 Agricultural Institute, Centre for Agricultural Research, Hungarian Academy of Sciences, Department of Plant Genetic Resources, H-2462 Martonvásár, Brunszvik u. 2, Hungary

**Keywords:** multicolour GISH, Thinopyrum intermedium, Thinopyrum ponticum, Agropyron glael, J, J^st^, St genomes

## Abstract

Multicolour genomic *in situ* hybridization (mcGISH) using total genomic DNA probes from *Thinopyrum bessarabicum* (Săvulescu & Rayss, 1923) Á. Löve, 1984 (genome J^b^ or E^b^, 2*n* = 14), and *Pseudoroegneria spicata* (Pursh, 1814) Á. Löve, 1980 (genome St, 2*n* = 14) was used to characterize the mitotic metaphase chromosomes of a synthetic hybrid of *Thinopyrum intermedium* (Host, 1805) Barkworth & D.R. Dewey, 1985 and *Thinopyrum ponticum* (Podpěra, 1902) Z.-W. Liu et R.-C.Wang, 1993 named „Agropyron glael” and produced by N.V. Tsitsin in the former Soviet Union. The mcGISH pattern of this synthetic hybrid was compared to its parental wheatgrass species. Hexaploid *Thinopyrum intermedium* contained 19 J, 9 J^St^ and 14 St chromosomes. The three analysed *Thinopyrum ponticum* accessions had different chromosome compositions: 43 J + 27 J^St^ (PI531737), 40 J + 30 J^St^ (VIR-44486) and 38 J + 32 J^St^ (D-3494). The synthetic hybrid carried 18 J, 28 J^St^ and 8 St chromosomes, including one pair of J-St translocation and/or decreased fluorescent intensity, resulting in unique hybridization patterns. Wheat line Mv9kr1 was crossed with the *Thinopyrum intermedium* × *Thinopyrum ponticum* synthetic hybrid in Hungary in order to transfer its advantageous agronomic traits (leaf rust and yellow rust resistance) into wheat. The chromosome composition of a wheat/A.glael F_1_ hybrid was 21 wheat + 28 wheatgrass (11 J + 14 J^St^+ 3 S). In the present study, mcGISH involving the simultaneous use of St and J genomic DNA as probes provided information about the type of *Thinopyrum* chromosomes in a *Thinopyrum intermedium*/*Thinopyrum ponticum* synthetic hybrid called A. glael.

## Introduction

N.V. Tsitsin produced a synthetic hybrid in the former Soviet Union by crossing *Thinopyrum intermedium* (Host, 1805) Barkworth & D.R. Dewey, 1985 (=*Agropyron glaucum* Roemer & Schultes, 1817, 2n=6x=42) with *Thinopyrum ponticum* (Podpěra, 1902) Z.-W.Liu & R.-C.Wang, 1993 (=*Agropyron elongatum* Host ex P. Beauvois, 1812, 2n=10x=70) (Tsitsin 1954). The hybrid plants were named “Agropyron glael” (A. glael, 2n=8x=56, Tsitsin 1979), from an abbreviation of “glaucum” and “elongatum”. This name (A. glael) will be used hereafter in this article. A number of A. glael plants were maintained in Martonvásár (Hungary) thanks to cooperation between the Hungarian Academy of Sciences and the Moscow Research Institute of Agriculture -“Nemchinovka” in the 1960’s. The hybrid plants had 56 chromosomes.

Both wheatgrass species are long been known to have superior resistance to various diseases (Wang 2011). They can be crossed with wheat, making them a potential source of gene pool for wheat improvement. In 2001, wheat line Mv9kr1 was crossed with A. glael in Hungary in order to transfer its advantageous agronomic traits (leaf rust and yellow rust resistance) into wheat (Molnár-Láng et al. 2012).

Polyploid *Thinopyrum* (Á. Löve, 1980) species contain genomes similar to the J (E^b^, J^b^) genome of the diploid *Thinopyrum bessarabicum* (Săvulescu & Rayss, 1923) Á. Löve, 1984 (2n=2x=14) (Östergen 1940) or the E (E^e^, J^e^) genome of *Thinopyrum elongatum* (Host, 1802) D.R. Dewey, 1984 (2n=2x=14) (Cauderon and Saigne 1961), which are closely related (Ceoloni et al. 2014), and sometimes also contain a third genome (S or St) from *Pseudoroegneria spicata* (Pursh, 1814) Á. Löve, 1980 (2n=2x=14). The S genome of *Pseudoroegneria* (Nevski, 1934) genus was renamed to St in order to discriminate from the S genome of Sitopsis section of *Aegilops* Linnaeus, 1753 species (Wang et al. 1995). Ceoloni (2014) also mentioned this genome as St/S. *Thinopyrum intermedium* has been described using various genome formulas, including E^e^E^b^St (Wang and Zhang 1996), E^1^E^2^St (Zhang et al. 1996) and JJ^s^S (Chen et al. 1998). Wang et al. (2011) mentioned J^s^ as E^St^ (J^St^). J^St^ symbol will be used hereafter to describe this special chromosome type of *Thinopyrum intermedium*. Kishii et al. (2005) and Mahelka et al. (2011) revealed new aspects of its genomic composition, suggesting the possible presence of a *Dasypyrum* (Cosson & Durieu de Maisonneuve, 1855) T. Durand, 1888 (V) genome. Recently Wang et al. (2015) published genotypic data obtained using EST-SSR primers derived from the putative progenitor diploid species *Pseudoroegneria spicata*, *Thinopyrum bessarabicum* and *Thinopyrum elongatum*, ﻿which indicated that the V genome was not one of the three genomes in intermediate wheatgrass. They proposed the J^vs^J^r^St genome designation, where J^vs^ and J^r^ represented ancestral genomes of the present-day J^b^ of *Thinopyrum bessarabicum* and J^e^ of *Thinopyrum elongatum*, J^vs^ being the more ancient. The change of J^s^ to J^vs^ is based on the study of Mahelka et al. (2011) and Deng et al. (2013), as all 14 chromosomes of J^s^ showed GISH/FISH hybridization signals from V-genome probes [*Dasypyrum villosum* (Linnaeus, 1753) P. Candargy, 1901], but only 8 to 11 of the 14 chromosomes have the centromeric region being hybridized by the St genome probe (Chen et al. 1998, Zhang et al. 1996, Kishii et al. 2005, Tang et al. 2011, Deng et al. 2013). FISH analysis using pMD232-500 as probe (originating from *Secale cereale* Linnaeus, 1753 cv. Kustro) indicated that the 14 J chromosomes of *Thinopyrum intermedium* bear FISH signals. According to their findings the J genome is changed to J^r^.The genome constitution of *Thinopyrum ponticum* was described using the JJJJ^s^J^s^ (Chen et al. 1998) and E^e^E^b^E^x^StSt (Li and Zhang 2002) formulas.

Genomic *in situ* hybridization (GISH) or multicolour genomic *in situ* hybridization (mcGISH) offered new opportunities for testing genome relationships in plants (Bennett et al. 1991), for describing hybrid character (Keller et al. 1996), for visualizing genomes simultaneously (Mukai et al. 1993), and for studying genome organization and evolution (Chen et al. 1994, Mahelka et al. 2011).

Multicolour genomic *in situ* hybridization was used in the present study for the simultaneous visualization of the J and St genomic DNA of A. glael and their parental wheatgrass species (*Thinopyrum intermedium*, *Thinopyrum ponticum*) and to describe the chromosome composition of these materials. As previously published papers had different findings and the authors proposed different genome formulas in *Thinopyrum intermedium*, difficulties in identification of the different genomes were expected in our study. As *Thinopyrum ponticum* chromosomes belonged to two different genomes (J and J^St^), precise detection and identification of them was probable despite of the high chromosome number. There were no former molecular cytogenetic data about the A. glael, but the presence of all the three different chromosome types (J, J^St^, St) of the two parental wheatgrass species was hoped-for.

## Methods

*Thinopyrum intermedium*, *Thinopyrum ponticum*, their synthetic hybrid A.glael, and the wheat/A. glael F_1_ hybrid were analysed cytogenetically (Table 1). Seeds of A.glael, wheat/A.glael F_1_ hybrid, *Thinopyrum intermedium*, and *Thinopyrum ponticum* were germinated, after which mitotic metaphase chromosome spreads were prepared according to Lukaszewski et al. (2004). McGISH was performed in order to simultaneously visualize the different wheatgrass chromosomes in *Thinopyrum intermedium*, *Thinopyrum ponticum*, A.glael, and in the Mv9kr1/A. glael F_1_ hybrid. J (E^b^) genomic DNA from *Thinopyrum bessarabicum* labelled with biotin-11-dUTP (Roche Diagnostics, Mannheim, Germany) and St genomic DNA from *Pseudoroegneria spicata* labelled with digoxigenin-11-dUTP were produced using the random primed labelling protocol. The hybridization mixture contained 100 ng each of the labelled probes/slide, dissolved in a 15 μl mixture of 100% formamide, 20×SSC and 10% dextran-sulphate at a ratio of 5:1:4, and 3000 ng *Triticum aestivum* (Linnaeus, 1753) DNA (genotype Mv9kr1, BBAADD) as a block when needed. Hybridization was performed at 42°C overnight. Streptavidin-FITC (Roche) and Anti-Digoxigenin-Rhodamine (Roche) dissolved in TNB (Tris-NaCl-blocking buffer) were used in the detection phase.The slides were screened using a Zeiss Axioskop-2 fluorescence microscope equipped with filter sets appropriate for DAPI (Zeiss Filterset 01), and for the simultaneous detection of FITC and Rhodamine (Zeiss filter set 24). Images were captured with a Spot CCD camera (Diagnostic Instruments) and processed with Image Pro Plus software (Media Cybernetics).

**Table 1.** Species and genotypes analysed in the present study.

Genotype	Accession number	Genebank	Geographic origin
*Thinopyrum intermedium*	PI565004	USDA ARS GRIN	Russia
*Thinopyrum ponticum*	PI 636523	USDA ARS GRIN	Argentina
*Thinopyrum ponticum*	PI531737	USDA ARS GRIN	Argentina
*Thinopyrum ponticum*	PI 547313	USDA ARS GRIN	Russia
*Thinopyrum intermedium* × *Thinopyrum ponticum* synthetic hybrid: Agropyron glael	glael-8/2008	Martonvásár Cereal Genebank	Russia
Mv9kr1 × A. glael F_1_ hybrid	112705	Martonvásár Cereal Genebank	Hungary

## Results

### *Thinopyrum intermedium*

**Figure 1.**
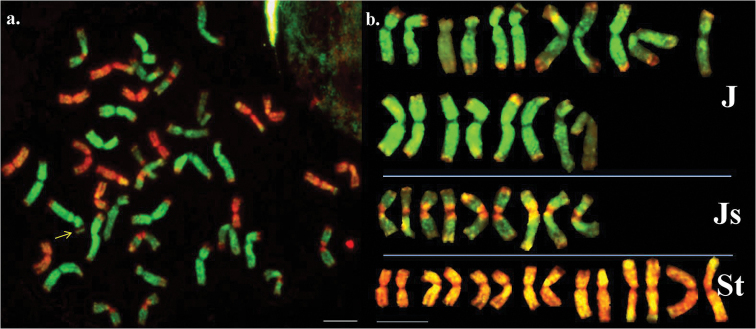
Results of multicolour genomic *in situ* hybridization on *Thinopyrum intermedium*. **a** Karyotype of a complete cell using *Thinopyrum bessarabicum* (J, green) and *Pseudoroegneria spicata* (St, red) genomic DNA as probes. Chromosome with satellite is indicated with arrow **b** Karyogram of *Thinopyrum intermedium* chromosomes. Top row: J chromosomes; middle row: J^St^ chromosomes with the St pericentromeric region; bottom row: St chromosomes. Bar = 10 μm.

McGISH, performed using J and St genomic DNA probes, simultaneously discriminated three different genomes in the segmental autoallohexaploid *Thinopyrum intermedium* (Fig. 1a–b). Among the 42 chromosomes, 14 fluoresced bright red along their whole length, showing the presence of the St genome. The St probe gave also a hybridization signal in the centromeric region of 9 chromosomes, where the other parts hybridized with the J genome, resulting in two-coloured chromosomes with a bow-tie shape. These J^St^-type chromosomes differed to those of the J, where the chromosomes hybridized with the J genomic DNA probe over the entire length with no centromeric St signals. The intensity of the green fluorescence signal was not uniform, the J^St^ chromosomes being fainter than J. In the telomeric segment of some J and J^St^ chromosomes a weak St genomic hybridization signal was detected, although in a few other chromosomes this fragment was unlabelled. One satellited chromosome was observed where the NOR region was hybridized to St genomic DNA. The analysed accession (No. PI565004) contained 19 J, 9 J^St^ and 14 St chromosomes.

### *Thinopyrum ponticum*

**Figure 2.**
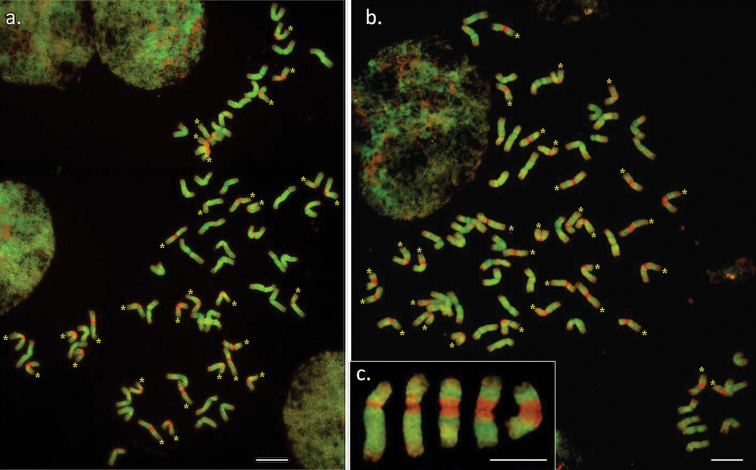
Multicolour genomic *in situ* hybridization on *Thinopyrum ponticum*. **a** Karyotype of *Thinopyrum ponticum* (accession VIR-44486) carrying 40 J and 30 J^St^chromosomes, using *Thinopyrum bessarabicum* (J, green) and *Pseudoroegneria spicata* (St, red) genomic DNA as probes **b** 38 J and 32 J^St^chromosomes identified in *Thinopyrum ponticum* (accession D-3494) **c** J^S^ chromosomes with different lengths of St DNA in the centromeric region. J^St^ chromosomes were marked with asterisks. Bar = 10 μm.

The analysed *Thinopyrum ponticum* contained 70 chromosomes and two groups could be distinguished based on their mcGISH pattern (Fig. 2). Bright green fluorescence signals marked the J chromosomes, while those with St (red) pericentromeric regions belonged to the J^St^ genome. The three analysed accessions showed different chromosome compositions: 43 J + 27 J^St^ (PI531737), 40 J + 30 J^St^ (PI 547313, Fig. 2a) and 38 J + 32 J^St^ (PI636523, Fig. 2b). The length of the St segment in the J^St^ chromosome varied (Fig. 2c). Each J^St^ chromosome showed a short section of St hybridization close to the centromere, while others fluoresced bright red on almost 1/3 of the chromosomes in the centromeric-pericentromeric regions. There was variation in the intensity of the green fluorescence signal, J^St^ chromosomes being fainter than J. The telomeric region of most of the chromosomes did not hybridize with the J or St genomic DNA probes and remained unlabelled.

### *Thinopyrum intermedium* × *Thinopyrum ponticum synthetic hybrid*: A. glael

**Figure 3.**
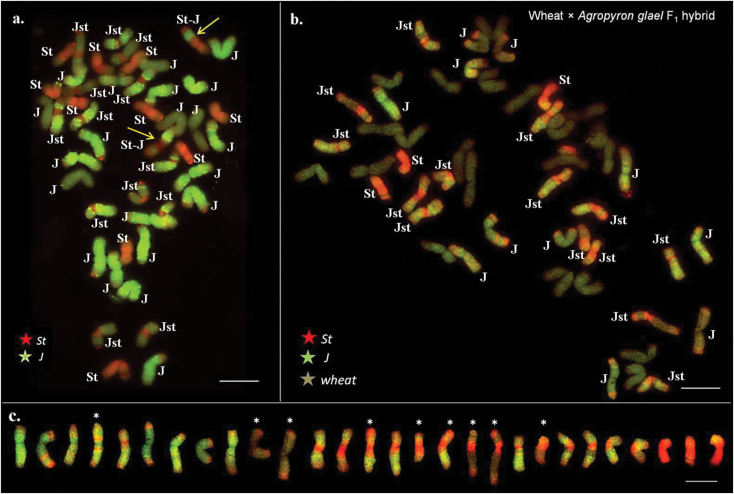
Multicolor genomic *in situ* hybridization pattern of Agropyron glael and the wheat (Mv9kr1 genotype)/A. glael F1 hybrid. **a** Karyotype of a partial cell of A. glael using *Thinopyrum bessarabicum* (J, green) and *Pseudoroegneria spicata* (St, red) DNA probes. Translocation between J and St chromosomes were marked with arrows **b** Karyotype of a complete cell of wheat (Mv9kr1 genotype)/A. glael F_1_ hybrid using *Thinopyrum bessarabicum* (J, green) and *Pseudoroegneria spicata* (St, red) genomic DNA simultaneously as probes and wheat genomic DNA as block simultaneously **c** Karyogram of A.glael chromosomes present in the wheat/A.glael F_1_ hybrid. Nine A. glael chromosomes with hybridization patterns different to their parental species are marked with asterisks. Bar = 10 μm.

McGISH made it possible to discriminate three different groups of A. glael chromosomes (Fig. 3a). The designation of the A. glael chromosomes was J, J^St^ and St, as the synthetic hybrid contains chromosomes from both *Thinopyrum intermedium* and *Thinopyrum ponticum*. Digoxigenin-labelled St genomic DNA hybridized to four pairs of submetacentric chromosomes, which were thus identified belonging to the St genome. One pair of chromosomes was mainly red, but an St-J translocation was detected in the long arm (marked with yellow arrowheads). Nine pair of chromosomes with only green fluorescence signals were identified as J genome, though three pairs showed lower fluorescence intensity, while the others were bright. The remaining fourteen pairs had various lengths of St genomic hybridization in the pericentromeric region, showing the presence of the J^St^ genome.

### Wheat/A. glael F_1_ hybrid

Chromosome counting detected 49 chromosomes in the wheat/A. glael F_1_ hybrid (21 wheat + 28 wheatgrass), 28 of which hybridized with the J and/or St genomes during mcGISH, discriminating the wheatgrass chromosomes from the unlabelled wheat (Fig. 3b). Only three chromosomes hybridized with the St genomic DNA over their entire length. Eleven chromosomes had no red fluorescence signal in the centromeric region, and were thus identified as J. Two of them had only very weak J signals, with stronger St hybridization in the telomeric region. The remaining 14 chromosomes had various lengths of St genomic hybridization in the pericentromeric region, showing the presence of the J^St^ genome. Some of the J and J^St^ chromosomes had a weak St genomic hybridization signal in the telomeric region. Some of these chromosomes carried several J-St, J^St^-St, translocations and/or decreased fluorescent intensity was observed in the pericentromeric and telomeric regions, resulting in unique hybridization patterns (marked with asterisks in Fig. 3c). The chromosome composition of the F_1_ hybrid was 21 wheat + 11 J + 14 J^St^+ 3 S.

## Discussion

GISH or mcGISH, a modification of fluorescence *in situ* hybrization, has been used to characterize genomes and chromosomes in polyploid *Thinopyrum* species (Chen et al. 1998, Tang et al. 2000, Li and Zhang 2002, Mahelka et al. 2011). In the present study, mcGISH involving the simultaneous use of St and J genomic DNA as probes provided information about the number and type of *Thinopyrum* chromosomes and demonstrated the presence of intergenomic (J-St) chromosome rearrangements in A. glael.

Chen et al. (1998) used GISH with one labelled genomic DNA probe and one nonlabelled blocking genomic DNA during the characterization of these wheatgrass species. They proposed the symbol J^S^ to represent J chromosomes with St repeated sequences and GISH signals around the centromeric regions. This chromosome type was the same which has been described in this study using two labelled genomic DNA probes. The use of mcGISH enabled the J and J^St^ genomes of *Thinopyrum ponticum* and the J, J^St^ and St genomes of *Thinopyrum intermedium* to be precisely discriminated using J and St labelled genomic DNA simultaneously.

As the number of J and J^St^ chromosomes was usually odd [19 J + 9 J^St^ in *Thinopyrum intermedium* and 43 J + 27 J^St^ (PI531737) in *Thinopyrum ponticum*], ﻿it is possible that J-J^St^ chromosome pairing can occur in meiosis, as reported by Chen et al. (2001). Most of the wheatgrass chromosomes were typical *Thinopyrum* chromosomes in A. glael and in the wheat/A. glael F_1_ hybrid, while others showed notable differences when the mcGISH patterns were compared to those of *Thinopyrum ponticum* and *Thinopyrum intermedium*: decreased fluorescence intensity, J-St translocations in the telomeric region of J^St^ chromosomes, and unlabelled chromosome parts in all types of chromosomes. Chen et al. (2001) reported a high frequency of chromosome pairing between J-J^St^, J-St and J^St^-St chromosomes, as the result of which genetic exchange is possible between these genomes. Several minor J-St and J^St^-St translocations were observed in A. glael and the wheat/A.glael F_1_ hybrid. These translocations may have occurred during the formation of the synthetic hybrids. As the J-J^St^-St chromosomes paired at high frequency, it may be that A. glael is not only a hybrid of the two wheatgrass species, but that the genetic composition has changed or been enriched with DNA sequences from other species during the long maintenance period (decades), as wheatgrass species are open-pollinating and very polymorphic. This could explain the presence of different hybridization patterns between the wheatgrass chromosomes in A. glael and the wheat/A.glael F_1_ hybrid.

Several types of genome composition and chromosome numbers have been reported for *Thinopyrum intermedium* (Chen et al. 1998, Tang et al. 2000, Da Yong et al. 2004). Chen et al. (1998) detect 41 chromosomes (18 J, 10 J^St^, 13 St) in a line derived from Portugal (PI249145), 49 chromosomes (18 J, 10 J^St^, 21 St) in a French genotype. Most of the analyzed accessions carried 42 chromosomes, but the number of each chromosome type was various: 20 J + 8 J^St^ + 14 St in ’Chef’, ’Clarke’ (USA), 18 J + 10 J^St^ + 14 St in PI317406 (Afghanistan), and 21 J + 7 J^St^ + 14 St in PI547333 (China) (Chen et al. 1998). Tang et al. (2000) analyzed a Chinese accession and identified 21 J + 7 J^St^ + 14 St chromosomes. Da Yong et al. (2004) could detect 28 J + J^St^ and 14 St chromosomes in PI 469214 (USA), PI 578698 (Russia), and Z1141 (Canada). In this study we could detect 42 chromosomes including 19 J, 9 J^St^ and 14 St. According to other findings and our results, when the chromosome number was not 42, the number of St chromosomes was derived. The number of J + J^St^ chromosomes was always 28. Mahelka et al. (2011) detected 42 chromosomes in different *Thinopyrum intermedium* accessions, and 14 of which hybridized with *Dasypyrum villosum* genomic DNA, and also carried St genomic DNA hybridization signal in the pericentromeric region. Mahelka et al. (2011) concluded that the genomic heterogeneity of intermediate wheatgrass was higher than had been assumed, making this species more interesting as a source of desirable agronomic traits.

Nucleolar dominance, an epigenetic phenomenon in which one parental set of ribosomal RNA (rRNA) genes is silenced in an interspecific hybrid or during allopolyploidization, first reported in the 1930s (Navashin 1934). Only ribosomal RNA genes inherited from one parent are transcribed (Pikaard 2000), and the nucleolus organiser regions (NORs), the sites of rRNA genes from the other parent(s) are suppressed. The phenomenon was observed in several interspecific hybrids (Gautam et al. 2014), including wheat/*Thinopyrum elongatum* addition lines (Linc et al. 2012). *Thinopyrum intermedium* and *Thinopyrum ponticum* are allopolyploid species, thus nucleolar dominance can probably be observed in them, and especially in their synthetic hybrid (A.glael). Loss of secondary constrictions can be observed during allopolyploidization or the formation of interspecific hybrids, which can be studied using rDNA probes by FISH. It was not part of this study, but it is planned in the future. Allopolyploidy can induce rapid genome evolution, and can cause genomic shock. The nature of this phenomenon were investigated (Matsuoka 2011). Its manifestation icludes chromosomal rearrangement, the gain and loss of chromosome segments, gene repression and activation, subfunctionalization, transposon activation, and changes in the epigenome (Wang et al. 2014). Multicolour GISH is a powerful technique to detect interspecific and intergeneric chromosome rearrangement. According to our mcGISH results, we observed minor chromosomal rearrangement, St-J translocations in nine chromosomes of A.glael in the wheat/ A.glael F1 hybrid, which chromosome patterns couldn’t observed in *Thinopyrum* parental species. The reduction of number of St chromosomes were also detected.

As A. glael contains chromosomes from the two most valuable *Thinopyrum* species, changes in its genome could result in new invaluable genetic material, especially for wheat breeding.

## Conclusions

In the present study, mcGISH involving the simultaneous use of St and J genomic DNA as probes provided information about the genome composition and the type of *Thinopyrum* chromosomes in a *Thinopyrum intermedium*/*Thinopyrum ponticum* synthetic hybrid called A. glael.
